# Monopolar Spindle 1 Kinase (MPS1/TTK) mRNA Expression is Associated with Earlier Development of Clinical Symptoms, Tumor Aggressiveness and Survival of Glioma Patients

**DOI:** 10.3390/biomedicines8070192

**Published:** 2020-07-03

**Authors:** Almuth F. Kessler, Jonas Feldheim, Dominik Schmitt, Julia J. Feldheim, Camelia M. Monoranu, Ralf-Ingo Ernestus, Mario Löhr, Carsten Hagemann

**Affiliations:** 1 Tumorbiology Laboratory, Department of Neurosurgery, University of Würzburg, Josef-Schneider-Str. 11, D-97080 Würzburg, Germany; Kessler_A1@ukw.de (A.F.K.); Jonas.Feldheim@googlemail.com (J.F.); Schmitt_D8@ukw.de (D.S.); Feldheim.Julia@gmail.com (J.J.F.); Ernestus_R@ukw.de (R.-I.E.); Loehr_M1@ukw.de (M.L.); 2 Department of Neuropathology, Intsitute of Pathology, University of Würzburg, Josef-Schneider-Str. 2, D-97080 Würzburg, Germany; camelia-maria.monoranu@mail.uni-wuerzburg.de

**Keywords:** glioblastoma multiforme, low-grade glioma, astrocytoma, recurrence, multifocal growth, mRNA expression, MPS1, TTK, therapy

## Abstract

Inhibition of the protein kinase MPS1, a mitotic spindle-checkpoint regulator, reinforces the effects of multiple therapies against glioblastoma multiforme (GBM) in experimental settings. We analyzed *MPS1* mRNA-expression in gliomas WHO grade II, III and in clinical subgroups of GBM. Data were obtained by qPCR analysis of tumor and healthy brain specimens and correlated with the patients’ clinical data. *MPS1* was overexpressed in all gliomas on an mRNA level (ANOVA, *p* < 0.01) and correlated with tumor aggressiveness. We explain previously published conflicting results on survival: high *MPS1* was associated with poorer long term survival when all gliomas were analyzed combined in one group (Cox regression: t < 24 months, *p* = 0.009, Hazard ratio: 8.0, 95% CI: 1.7–38.4), with poorer survival solely in low-grade gliomas (LogRank: *p* = 0.02, Cox regression: *p* = 0.06, Hazard-Ratio: 8.0, 95% CI: 0.9–66.7), but not in GBM (LogRank: *p* > 0.05). This might be due to their lower tumor volume at the therapy start. GBM patients with high *MPS1* mRNA-expression developed clinical symptoms at an earlier stage. This, however, did not benefit their overall survival, most likely due to the more aggressive tumor growth. Since *MPS1* mRNA-expression in gliomas was enhanced with increasing tumor aggressiveness, patients with the worst outcome might benefit best from a treatment directed against MPS1.

## 1. Introduction

Glial tumors encompass a group of primary brain tumors that are classified into different subgroups by the World Health Organization (WHO) [1]. The natural history of these tumors varies greatly. Pilocytic astrocytomas WHO grade I (PA) represent a benign form, mainly found in children, with a 10-year survival of over 90% [2,3,4]. In comparison, low-grade astrocytomas WHO grade II and III grow faster and more infiltrative. Formerly, these tumors were mainly classified due to their histological behavior. With the recent update of the WHO classification, however, molecular markers are now considered as well [1]. For instance, the diagnosis ”gliomatosis cerebri” no longer exists, as those widespread tumors with infiltration of three or more lobes show a methylation profile comparable to the other glial tumor entities [5]. *Isocitrate dehydrogenase* (IDH) mutant gliomas of WHO grade II or III (IDHmut glioma) are a slowly growing subcategory, with a comparatively good prognosis.

In contrast, *IDH*-wildtype gliomas of WHO grade II and III (IDHwt glioma), show a growth pattern and patients’ clinical course that resembles those of glioblastoma multiforme (GBM), and therefore might even be an early form of a GBM [6,7,8]. GBM is not only the most common but also the most aggressive form of primary brain tumor [9]. The current standard therapy, consisting of tumor resection, irradiation, concomitant temozolomide (TMZ) chemotherapy, and adjuvant TMZ-treatment, was established in 2005, with a median patient survival of only 14.6 months [10].

After 12 years with mainly negative clinical phase III trials, Tumor Treating Fields (TTFields) raised new hopes for an improvement of the standard therapy [11,12,13]. Recently, we demonstrated that the effects of TTFields are augmented and accelerated by mitotic checkpoint inhibition of the protein kinase monopolar spindle 1 (MPS1, also known as TTK) in vitro [14], indicating the potential for a combined treatment advantage. MPS1-inhibition reduces cell proliferation of GBM when combined with the antimitotic agent vincristine in vitro and in vivo [15]. It is a dual-specificity protein kinase that regulates the mitotic spindle checkpoint by monitoring proper chromosome alignment and attachment to spindle microtubuli [16,17,18,19,20,21]. Dysregulation of MPS1 activity has been reported to lead to chromosomal instability and cancerogenesis [19]. It is overexpressed in astrocytic tumors, with overexpression correlating with tumor grade and patients’ survival [15]. However, to our knowledge, there are no data published on astrocytic tumors of different growth patterns or on glioma recurrence. Interestingly, aberrant *MPS1* expression and its effects appear to be hugely influenced by their dysregulation of *MPS1* mRNA and its regulator miR-132 [22]. Therefore, it appears promising to explore connections between *MPS1* mRNA expression and patients’ clinical course, to lead the way from experimental observations to a translational implementation into clinical research and hopefully, ultimately, patient care. The purpose of this study was (1) to confirm and extend the qPCR-results reported by us previously [15] and (2) to provide information on the expression of *MPS1* in gliomas of different biological behavior and clinical course on an mRNA level. We show that *MPS1* mRNA was indeed dysregulated and overexpressed in gliomas, associated with earlier development of patients’ clinical symptoms, correlated to tumor aggressiveness, and associated with the survival of patients with low-grade gliomas.

## 2. Experimental Section

### 2.1. Tissue Samples and Clinical Data

All included patients were treated at the Department of Neurosurgery, University Hospital Würzburg, University of Würzburg. All subjects gave their informed consent for inclusion before they participated in the study. The study was conducted in accordance with the declaration of Helsinki, and the protocol was approved on 16-July-2014 by the Institutional Review Board of the University of Würzburg (#103/14). After gaining the tissue during surgery, equal shares were frozen in liquid nitrogen for molecular analyses and embedded in paraffin for immunohistochemistry. The tumors were classified by routine histology in accordance with WHO criteria [1]. Only tumor samples with a typical histological appearance according to the evaluation by an experienced neuropathologist and originating from central, viable tumor areas were included. In addition, GBM samples with an estimated tumor cell content of less than 80% were excluded. The Brain Bank of the Department of Neuropathology, Institute of Pathology, University of Würzburg, Germany was the source for autopsy/biopsy samples of non-pathological brain tissue (normal brain, NB) (approval #78/99 from 09-July-1999 and 04-October-2016). Clinical information of the patients such as tumor localization and growth (e.g., local or multifocal), treatment modalities, recurrence, and outcome were collected retrospectively (Table 1). The tumor volume and extent of surgical resection were measured by evaluating pre- and post-operative Magnetic Resonance Imaging (MRI) images. The IDH mutation status was determined by immunohistochemistry and pyrosequencing, the Ki67 status by immunohistochemistry and the *MGMT* promoter methylation status by high resolution melting real-time polymerase chain reaction. Since some of the specimens were already analyzed for a previous project, the methodology has been described in detail elsewhere [23,24].

### 2.2. RNA Extraction and qPCR

mRNA was extracted from frozen tissue samples by the TRIzol^®^ Reagent (Thermo Fisher Scientific, Waltham, MA, USA) and converted to DNA, as previously described [23]. qPCR (Quantitative Real-Time Polymerase Chain Reaction) was performed on a StepOnePlus Real-time PCR System (Applied Biosystems, Foster City, CA, USA) to determine the *MPS1* mRNA expression in a duplex setting utilizing the TaqMan Universal PCR Master Mix, TTK_FAM (Hs01009870_m1,) and GAPDH_VIC_PL (Hs99999905_m1) (all from Applied Biosystems, Foster City, CA, USA) according to the manufacturer’s instruction. Each sample was tested in technical triplets with 20 ng cDNA each. The PCR’s cycling started at 50°C for 2 min, followed by enzyme activation at 95°C for 10 min and 50 cycles of 15 s at 95°C and 1 min at 60°C. If the technical replicates exceeded a standard deviation of 0.5 Cq, the qPCR was repeated. Cq-values were adjusted to a relative standard curve following the manufacturer’s instructions.

### 2.3. Statistics

The analyses were based on the ΔΔCq-values, which were directly obtained from the qPCR [25] and normalized to the expression of the housekeeping gene *Glyceraldehyde 3-phosphate dehydrogenase* (*GAPDH*). As autopsy- and biopsy-derived NB specimens had a similar expression, they were combined into one group. In addition, we analyzed *MPS1* mRNA expression data available from the IVY-GAP database [26] (https://glioblastoma.alleninstitute.org), accessed in March 2020. Statistical calculations were performed with IBM SPSS Statistics 23 (IBM Corporation, Armonk, NY, USA). Boxplots show the relative quantity calculated from the ΔΔCq-values. A comparison of expression and regression analysis was performed with ANOVA (Levene’s test, Post-hoc: Scheffe-test or Dunnet-T3), correlation analysis with Spearman’s Rho. The patients’ outcome was compared by Kaplan–Meier (LogRank), as well as cox regression on time-dependent variables. We refrained from the survival analysis of IDHwt gliomas due to low sample quantity.

## 3. Results

### 3.1. Patient Cohort

We assessed the MPS1 mRNA expression of 7 normal brain (NB) (epilepsy surgery: *n* = 3, autopsy: *n* = 4), 4 PA, 25 IDHmut glioma, 11 IDHwt glioma and 57 GBM specimens with different growth patterns at first diagnosis and relapse. Additionally, we retrospectively collected clinical data of 58 of these patients. Unfortunately, some patients were partly treated in external institutions, limiting access to their clinical information. A low amount of tumor material prevented us from performing all analyses with some samples. Therefore, information on the extent of tumor resection (*n* = 1), *MGMT* promoter methylation (*n* = 14), and Ki67 staining (*n* = 3) is missing for some patients. The available characteristics, molecular and prognostic parameters, outcome results, etc. are summarized in Table 1 (GBM) and Table 2 (IDHmut and IDHwt glioma).

### 3.2. MPS1 mRNA was Overexpressed in Gliomas and Correlated with Tumor Aggressiveness

**Figure 1:**
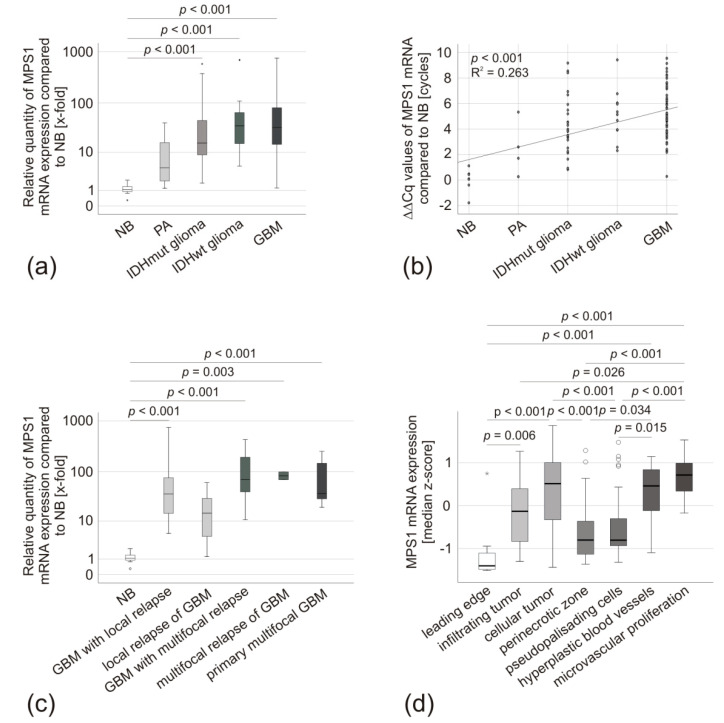
*MPS1* mRNA expression in glioma specimens. (**a**) Box-plot comparing *MPS1* mRNA expression in NB (*n* = 7), PA (*n* = 4, median expression 12-fold), IDHmut glioma (*n* = 25, median expression 73-fold), IDHwt glioma (*n* = 11, median expression 96-fold), and GBM (*n* = 57, median expression 91-fold). The median is displayed by the middle line. The quartiles are represented by the hinges, extreme values, up to 1.5 times the height of the box, are shown by whiskers and outliners represented by points. NB was set as the reference. Groups were compared by ANOVA with Levene’s test to assess the equality of variances and Scheffe procedure or Dunnet-T3 as post hoc tests. *p*-values are based on differences of ΔΔCq-values. (**b**) Scatter diagram of tumor aggressiveness and *MPS1* mRNA expression, linear regression, *p*-values were calculated by ANOVA based on ΔΔCq-values. The global effect size was determined by Cohen’s *f*. (**c**) Boxplot of *MPS1* mRNA expression in GBM with local relapse (n=14, median expression 112-fold), local relapse of GBM (*n* = 5, median expression 21-fold, *p* = 0.06), GBM with multifocal relapse (*n* = 8, median expression 130-fold), multifocal relapse of GBM (*n* = 2, median expression 84-fold), and primary multifocal GBM (*n* = 7, median expression 94-fold) compared to NB (*n* = 7). The analysis was performed as described in (a). (**d**) IVY-GAP database [26] analysis of *MPS1* mRNA expression in different areas of GBM: leading edge (*n* = 16), infiltrating tumor (*n* = 24), cellular tumor (*n* = 110), perinecrotic zone (*n* = 25), pseudopalisading cells around necrosis (*n* = 40), hyperplastic blood vessels in cellular tumor (*n* = 22), and microvascular proliferation (*n* = 28).

In comparison to NB, MPS1 mRNA was significantly overexpressed in IDHmut glioma (*p* < 0.001, median: 73-fold) IDHwt glioma (*p* < 0.001, median: 96-fold) as well as in GBM (*p* < 0.001, 91-fold). However, there was no significant difference in the *MPS1* mRNA expression between these three groups (*p* > 0.05) (Figure 1a and Table 3). Benign PA displayed a statistically non-significant tendency towards overexpression (Figure 1a). Nevertheless, by sorting these entities according to their malignancy, a highly significant correlation of MPS1 mRNA expression and tumor aggressiveness was detectable (*p* < 0.001, R^2^ = 0.263) (Figure 1b). When we analyzed the different GBM-subgroups distinguished by their growth pattern (primary local tumors leading to local relapse, primary local tumors leading to multifocal relapse and primary multifocal tumors) and compared primary tumors and relapses, we did not observe any statistically significant difference in *MPS1* mRNA expression (*p* > 0.05) (Figure 1c and Table 3). Nevertheless, despite different growth and relapse patterns, all tumors displayed significant *MPS1* overexpression compared to NB (*p* < 0.001). Only the local relapses of GBM, although displaying a stronger expression, were not significantly different from NB (*p* = 0.06) (Figure 1c and Table 3). Similarly, both IDHmut glioma with local growth and IDHmut gliomas with highly diffuse growth (infiltration of three or more lobes, formerly classified as “gliomatosis cerebri”) had significantly overexpressed *MPS1* mRNA in comparison to NB (both *p* < 0.001), but not between each other (Table 3). The IVY Glioblastoma Atlas Project (IVC-GAP) database allows the examination of regional differences in mRNA expression within the tumor [26]. It revealed enhanced *MPS1* expression in the cellular tumor mass, hyperplastic blood vessels, and microvascular proliferation, but not in the leading edge, infiltrating tumor, the perinecrotic zone, or pseudopalisading cells around necrosis (Figure 1d).

### 3.3. MPS1 mRNA Expression Correlated with Earlier Development of Clinical Symptoms, Tumor Volume and Long Term Survival of Patients

**Figure 2:**
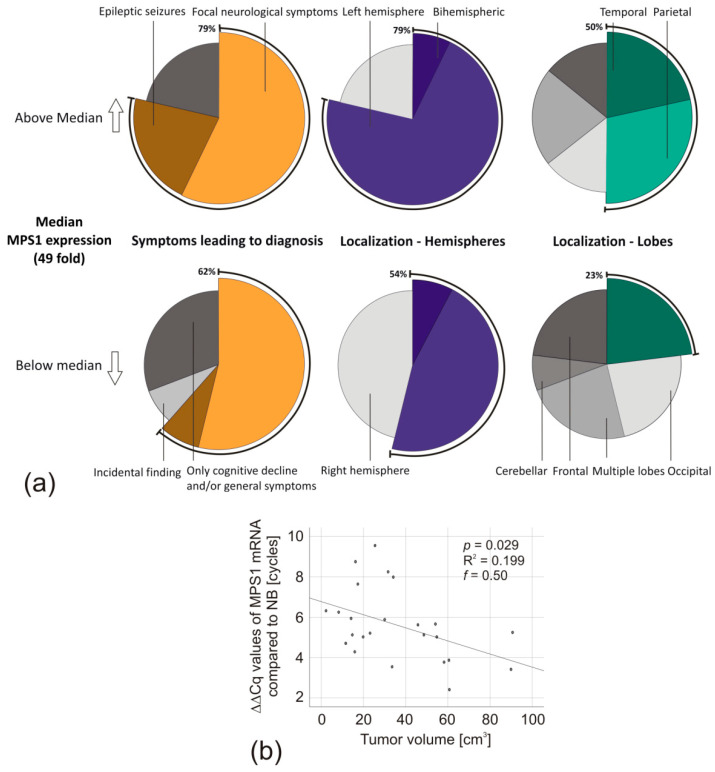
Association of *MPS1* mRNA expression with GBM-patients’ symptoms, tumor localization, and tumor volume. (**a**) Pie charts of the reason for initial hospitalization and tumor localization of GBM patients with *MPS1* mRNA expression below and above the median expression (49-fold). Shown is the percentage of patients belonging to each group. (**b**) Scatter diagram of *MPS1* mRNA expression compared to the GBM tumor volume. Linear regression, *p*-values were calculated by ANOVA based on ΔΔCq-values. The global effect size was determined by Cohen’s *f*.

Having confirmed that *MPS1* mRNA was generally overexpressed in gliomas and particularly in conjunction with glioma aggressiveness, we wondered whether the clinical course of the patients also reflected this. The median *MPS1* expression was 49-fold. Interestingly, when looking at the initial reasons for hospitalization (epileptic seizures, focal neurological symptoms, cognitive decline and/or general symptoms, or incidental finding), GBM patients with *MPS1* expression above this threshold more often had focal neurological symptoms or epileptic seizures (11 of 14, 79%), compared to those with *MPS1* expression below the median (8 of 13, 62%) (Figure 2a). In addition, their tumors were frequently localized solely in the left hemisphere (above median MPS1 expression: 10 of 14, 71%; below median MPS1 expression: 6 of 13, 46%), especially in the temporal or parietal lobe (above median *MPS1* expression: 7 of 14, 50%; below median *MPS1* expression: 3 of 13, 23%) (Figure 2a). In addition, GBM patients with *MPS1* mRNA expression below the median had a significantly higher tumor volume at diagnosis (45 vs. 25 cm^3^, *p* = 0.043, unpaired two-tailed *t*-test). This observation was confirmed by regression analysis. *MPS1* mRNA expression correlated negatively with the tumor volume (*p* = 0.029, R^2^ = 0.199, *f* = 0.50) (Figure 2b). Other clinical parameters did not show any correlation with MPS1 mRNA expression (Table 4). 

**Figure 3:**
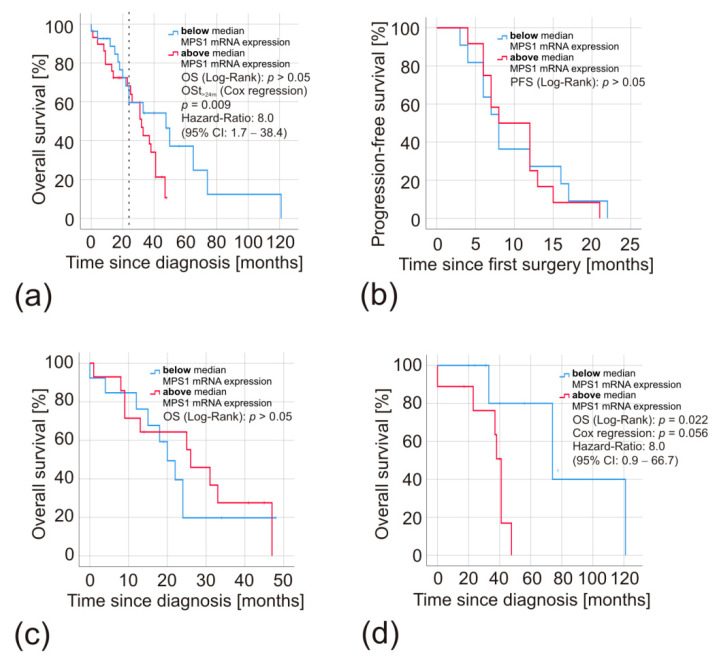
Kaplan–Meier analyses of patients with high and low *MPS1* mRNA expression in their gliomas. Survival of patients with *MPS1* mRNA expression below (**blue**) and above (**red**) the median expression of 49-fold was compared. Hazard ratios and time-dependent effects were calculated by applying the Cox proportional hazards model, *p*-values by the Log Rank test. (**a**) Overall survival of all glioma patients combined independently of their WHO grade (*MPS1* mRNA expression high *n* = 27, low *n* = 29). The dashed line indicates a survival time of 24 months. (**b**) Progression-free and (**c**) overall survival of GBM patients (*MPS1* mRNA expression high *n* = 12 and *n* = 14, respectively, low *n* = 11 and *n* = 13, respectively). (**d**) Overall survival of patients with IDHmut gliomas (*MPS1* mRNA expression high and low *n* = 9, each).

To assess patients’ survival, we combined all gliomas in one group independently of their WHO grading, as performed in a previous publication by Tannous et al. [15]. After separating them according to the median *MPS1* mRNA expression, there was no significant difference in the overall survival of patients exposed to Kaplan–Meier analysis (Figure 3a). However, closer examination revealed that both curves ran close to parallel for two years, before significantly diverging when it came to long-time survival (Cox regression: t < 24 months, *p* = 0.009, Hazard ratio = 8.0 (95% CI: 1.7–38.4)) (Figure 3a). The GBM subgroup analyses of the progression-free (Figure 3b) and overall survival (Figure 3c) indicated a comparable clinical course of the groups with *MPS1* mRNA expression above or below the median. Interestingly, IDHmut gliomas did not mirror this course. The IDHmut glioma patients with low *MPS1* mRNA expression had a significantly longer survival (LogRank: *p* = 0.02, Cox regression: *p* = 0.06, Hazard-Ratio: 8.0 (95% CI: 0.9–66.7) than those with high *MPS1* mRNA expression (Figure 3d).

## 4. Discussion

MPS1 has been reported to be overexpressed in glioma tissue on an mRNA level, increasing concomitantly with tumor malignancy [15,28,29], and shows potential as a therapeutic target for the treatment of central nervous system tumors [14,30,31,32]. So far, there are only few publications providing information about MPS1 expression in patients’ tissue [15,22,28,29,32]. Therefore, it was vital to confirm and extend those data to validate MPS1 as a future therapeutic target.

Bie et al. and Tannous et al. were the first to analyze *MPS1* mRNA expression in gliomas of different grades, revealing that *MPS1* mRNA was dysregulated and overexpressed in glial tumors grade II-IV [15,28]. Further, the extent of overexpression rose with increasing tumor malignancy [15,29]. Their observations are mirrored by Chen et al., who further suggest a dysregulation of the HLF/miR-132/MPS1 axis to be causal for the malignancy-dependent expression differences [22]. Alimova et al. reported similar results for 90 pediatric brain tumors [32]. *MPS1* mRNA was weakly expressed in cerebellar tissue, as well as cerebral and fetal brain tissue, while the malignant entities atypical teratoid rhabdoid tumors, GBM, and medulloblastomas displayed a high level of *MPS1* mRNA. Interestingly, the authors observed a tendency towards overexpression in benign pediatric PA. 

Our analyses confirm the previous observations. The malignant glial tumors strongly expressed *MPS1* mRNA, increasing concomitantly with tumor malignancy/aggressiveness, while the benign adult PA only mildly overexpressed *MPS1*. In the GBM subgroups, we did not detect significant differences between primary tumors and their respective relapses nor between tumors of different growth patterns. The same was true for IDHwt glioma and IDHmut glioma. Therefore, we conclude that the *MPS1* mRNA dysregulation in glial tumors is associated with tumor malignancy but occurs independently of recurrence or growth pattern. Interestingly, the IVY-GAP database [26] revealed that its expression was regionally enhanced in the cellular tumor mass, hyperplastic blood vessels, and microvascular proliferation. These cells are highly proliferative [9,33], while the cells in tumor areas with low *MPS1* expression, the leading edge, infiltrating tumor, the perinecrotic zone, and pseudopalisading cells are mainly migrating [34,35]. This expression pattern is in accordance with the role of MPS1 as a spindle assembly checkpoint regulator during the mitosis of cells [16,17,18,19,20,21].

Consequently, we wondered whether such association with proliferation and malignancy was also reflected by the patients’ clinical characteristics. We divided our panel by the median *MPS1* mRNA expression and could subsequently observe that patients with high expression were numerically more often hospitalized with epileptic seizures and focal neurological symptoms. In addition, their tumors were frequently localized in the left hemisphere, as well as the temporal and parietal lobe. In right-handed patients, who represent the majority in the population, the left hemisphere is dominant. The temporal and parietal lobes are known for critical neurological pathways and, among other areas, are responsible for the tactile sense and speech [36]. Therefore, lesions in these areas can lead to earlier and more prominent symptoms. This misdistribution could be a further indication of *MPS1* mRNA expression being associated with aggressive tumor growth. However, additional confirmation is required to rule out the possibility of a statistical coincidence.

Interestingly, patients with high expression had less than half of the tumor volume at initial diagnosis. As diagnosis predominantly occurs after symptoms of a certain severity (neurological deficits, epileptic seizures, changes of personality, etc.) led to hospitalization, this raises the question of whether *MPS1* overexpression might lead to more dominant and severe clinical deficits at an earlier stage. Since the tumor volume is a prognostic predictor, we investigated the patients’ survival.

Previous analyses on MPS1 correlation with patients’ survival resulted in contrasting conclusions. Tannous et al. describe a significant difference in survival between a group of high and intermediate MPS1 expression in gliomas of all WHO grades combined [15]. A similar observation was made by Wang et al. for *MPS1* mRNA and protein expression by analyzing the Rembrandt database and immunohistochemically stained tissue slides, respectively [29]. In contrast, Alimova et al. did not detect a difference in outcome in different pediatric high-grade tumor entities (GBM, medulloblastoma, atypical teratoid rhabdoid tumor) [32], an observation confirmed by Bie et al. for GBM from adult patients [28]. We aimed to evaluate these conflicting results.

To reproduce the Kaplan–Meier analysis performed by Tannous et al. [15], we combined all glioma patients and divided them by their median *MPS1* expression. Similarly to these authors, both curves ran almost parallel for approximately two years before they significantly separated, as confirmed by time-dependent cox regression (*p* = 0.009). However, this effect seems to be primarily based on a group of long-term survivors consisting of grade II/III glioma patients. While these low-grade glioma patients benefitted from low *MPS1* mRNA expression, there was no significant difference in the overall or progression-free survival of GBM patients. This observation confirms the results of Bie et al. and Alimova et al. and sheds light on the apparent contrast between these previous publications [15,28,32]. Remarkably, the survival time of the GBM patients was equal, although the group with low MPS1 expression at diagnosis had almost twice the tumor volume of the group with high expression. High pretreatment tumor volume at diagnosis of the primary tumor and relapse is usually associated with poorer survival [37,38,39]. This mismatch in tumor volume may provide another explanation for the significant effect in outcome when all tumor entities are analyzed together, while subgroup analyses provided negative results. All three previous reports did not consider the tumor volume as a prognostic factor [15,28,32].

To correctly interpret our results, it should be noted that the number of specimens in some subgroups is rather small (e.g., multifocal relapse of GBM), which restrained us from performing multivariable analyses. However, samples of some of the examined tumor sub-entities and data on the role of MPS1 in brain tumors in general, are scarce. Therefore, we consider our results highly valuable, notably, as we observed distinct and statistically significant differences in mRNA expression regardless of the limited number of samples.

Multiple sources have recently suggested MPS1-inhibition to be a viable treatment option for brain tumors, either alone [31,32] or in combination with other therapeutics such as radiation therapy [30], chemotherapy [15], or TTFields [14]. We report MPS1 overexpression in all analyzed tumor entities and subgroups. Therefore, all patients with glial tumors might potentially benefit from such combination therapies, independently of the growth pattern or patient characteristics. At least four different MPS1-inhibitors (BOS-172722, CFI-402257, S81694, and BAY-1217389) are currently tested in Phase I trials on various advanced malignancies [40]. Though the permeability of the blood–brain barrier of these drugs has not been reported in detail, the first preliminary results of their application look promising, and the authors declare good tolerability and manageable adverse effects [41]. Consequently, the inhibition of MPS1 might soon be generally available as a treatment option. 

MPS1 expression in gliomas is enhanced with increasing tumor aggressiveness. Therefore, we hypothesize that patients with gliomas WHO grade II/III and high MPS1 expression and especially glioma patients with the worst expected outcome might benefit most from a treatment directed against MPS1. As the dysregulation of MPS1 expression begins at the mRNA level, qPCR-based tests might help to further narrow the pre-selection of suitable patients.

## Figures and Tables

**Table 1:** Clinical parameters of glioblastoma multiforme (GBM) patients (*n* = 27).

**Patients’ characteristics**				
**Sex**	female: 10/37.0%		male: 17/63.0%	
**Age** (median, quartiles)	56 years (49–65 years)			
**ECOG**	0: 15/55.6%	1: 10/37.0%		>1: 2/7.4%
**Tumor characteristics**				
**Volume** (median, quartiles)	30.9 cm^3^ (16.0–54.6 cm^3^)			
**Localization**	left hemisphere: 16/59.3%	right hemisphere: 9/33.3%		both hemispheres: 2/7.4%
**Localization** (lobe)	frontal: 5/18.5%	occipital: 5/18.5%		temporal: 6/22.2%
	parietal: 4/14.8%	multiple: 6/22.2%		cerebellar: 1/3.7%
**IDH mutation status** ^1^	IDHwt: 21/87.5%		IDHmut: 3/12.5%	
***MGMT* promoter methylation** ^1^	unmethylated: 7/30.4%		methylated: 16/69.6%	
**Ki67 staining** (median, quartiles)	25% (20–30)			
**Therapy**				
**Time from diagnosis to therapy**	0–7 days: 18/66.7%	8–14 days: 5/18.5%		>14 days: 4/14.8%
**Surgical intervention** ^2^	biopsy: 4/15.4%	complete resection: 4/15.4%		incomplete resection: 18/69.2%
**Radiation therapy**	yes: 25/92.6%		no: 2/7.4%	
**TMZ chemotherapy**	yes: 22/81.5%		no: 5/18.5%	
**Relapse and Outcome**				
**PFS** ^3^	8 months (6–13 months)			
(median, quartiles)				
**Relapse**	primarily multifocal: 4/14.8%	local: 15/55.6%		multifocal: 8/29.6%
**Therapy in relapse** ^2^	surgical resection followed by radiationand /or TMZ: 12/52.2%	radiation and/or TMZ: 5/21.7%		best supportive care: 6/26.1%
**OS**	19 months (9–13 months)			
(median, quartiles)				

Absolute numbers and the respective percentages are shown. ^1^ Due to a lack of sufficient tissue samples, the IDH mutation and the *MGMT* promoter methylation status could not be re-evaluated for some patients. ^2^ For some patients partly treated in external institutions, information about therapeutic interventions was limited. ^3^ Some patients did not match the criteria for tumor progression and, therefore, were excluded from the PFS analysis. ECOG = Eastern Cooperative Oncology Group score [27]; IDH = isocitrate dehydrogenase; IDHmut = IDH-mutated tumors; IDHwt = IDH wildtype tumors; MGMT = O^6^-methylguanine-DNA methyltransferase; TMZ = Temozolomide; PFS = progression-free survival; OS = overall survival.

**Table 2:** Clinical parameters of IDHmut (*n* = 20) and IDHwt glioma (*n* = 11) patients.

Patients’ Characteristics	IDHmut Glioma	IDHwt Glioma
**Sex**	female: 6/30.0% male: 14/70.0%	female: 2/18.2% male: 9/81.8%
**Age** (median, quartiles)	37 years (33–45 years)	53 years (26–58 years)
**OS** (median, quartiles)	37.5 months (23.75–42.65 months)	32.0 months (14.0–48.0 months)
**Growth pattern**	local: 10/50.0% highly diffuse: 10/50.0%	local: 6/54.5% highly diffuse: 5/45.5%

Absolute numbers and the respective percentages are shown. The infiltration of fewer than three lobes was defined as local and the infiltration of three or more lobes as highly diffuse growth. OS = overall survival; IDH = isocitrate dehydrogenase; IDHmut glioma = IDH-mutated tumors with the histological appearance of WHO grade II and III gliomas; IDHwt gliomas = IDH wildtype tumors with the histological appearance of WHO grade II and III gliomas.

**Table 3:** Statistical data of the *MPS1* mRNA expression analysis.

NB Compared to:	Difference in ΔΔCq-Values (Median)	95%-CI	*p*-Value
PA	2.5	−1.4–6.4	0.42
IDHmut glioma	4.4	1.7–7.0	**<0.001**
IDHmut glioma with local growth	4.4	1.7–7.2	**0.001**
IDHmut glioma with highly diffuse growth	4.8	2.1–7.5	**<0.001**
IDHwt glioma	5.1	2.1–8.1	**<0.001**
GBM	5.3	2.8–7.8	**<0.001**
GBM with local relapse	5.5	2.7–8.2	**<0.001**
Local relapse of GBM	3.4	−0.1–6.9	0.06
GBM with multifocal relapse	6.3	3.2–9.4	**<0.001**
Multifocal relapse of GBM	6.4	1.6–11.2	**<0.001**
Primary multifocal GBM	5.9	2.8–9.1	**<0.001**

Groups were compared by ANOVA with Levene’s test to assess the equality of variances and Scheffe procedure or Dunnet-T3 as posthoc tests. P-values are based on differences of ΔΔCq-values. *p*-values below 0.05 are shown in bold. Local growth was defined as the infiltration of fewer than three lobes and highly diffuse growth as the infiltration of three or more lobes. NB = normal brain; CI = confidence interval; PA = pilocytic astrocytoma; IDH = isocitrate dehydrogenase; IDHmut glioma = IDH-mutated tumors with the histological appearance of WHO grade II and III gliomas; IDHwt gliomas = IDH wildtype tumors with the histological appearance of WHO grade II and III gliomas; GBM = glioblastoma.

**Table 4:** Correlation of patient and tumor characteristics with *MPS1* mRNA expression.

	IDHmut Glioma		GBM				
	Age (years)	OS (months)	Age (years)	OS (months)	PFS (months)	Tumor Volume (cm^3^)	Ki67 Staining (%)
**Correlation coefficient**	0.20	−0.01	0.37	−0.17	−0.27	**0.43**	−0.27
***p*-value**	0.41	0.96	0.06	0.40	0.21	**0.04**	0.19

Non-parametric tests (Spearman’s Rho) were utilized to correlate *MPS1* mRNA expression with the selected patient and tumor characteristics. Significant results are highlighted in bold. IDH = isocitrate dehydrogenase; IDHmut glioma = IDH-mutated tumors with the histological appearance of WHO grade II and III gliomas; GBM = glioblastoma; OS = overall survival; PFS = progression-free survival.

## Abbreviations

GBM	Glioblastoma
WHO	World Health Organization
IDH	Isocitrate dehydrogenase
IDHwt glioma	IDH-wild-type gliomas of WHO grade II and III
IDHmut glioma	IDH-mutated gliomas of WHO grade II and III
TMZ	Temozolomide
TTFields	Tumor Treating Fields
MPS1/TTK	Protein kinase Monopolar spindle 1
qPCR	Quantitative real-time polymerase chain reaction
mRNA	Messenger ribonucleic acid
NB	Normal brain
MGMT	O^6^-methylguanine-DNA methyltransferase
ECOG	Eastern Cooperative Oncology Group score
PFS	Progression-free survival
OS	Overall survival
ANOVA	Analysis of variance
PCR	Polymerase Chain reaction
GAPDH	Glyceraldehyde 3-phosphate dehydrogenase
MRI	Magnetic Resonance Imaging
